# Börjeson-Forssman-Lehmann Syndrome in a Pediatric Patient: A Four-Year Longitudinal Case Report Focused on Functional Evolution and Rehabilitation

**DOI:** 10.7759/cureus.101421

**Published:** 2026-01-13

**Authors:** Marco Francisco Silva, Vitor Machado Sousa, Raquel Sofia Santos, José Pedro Pimenta, Helena Reis Costa

**Affiliations:** 1 Physical Medicine and Rehabilitation, Unidade Local de Saúde do Alto Ave, Guimarães, PRT

**Keywords:** börjeson-forssman-lehmann syndrome, case-report, developmental disabilities, rehabilitation, x-linked neurodevelopmental disorders

## Abstract

Börjeson-Forssman-Lehmann syndrome (BFLS) is a rare X-linked neurodevelopmental disorder caused by pathogenic variants in the plant homeodomain finger protein 6 (PHF6) gene, typically associated with intellectual disability, hypotonia, dysmorphic features, endocrinological abnormalities, epilepsy, and global developmental impairment. Most published literature focuses on genetic and phenotypic characterization, with limited information regarding rehabilitation or long-term functional outcomes. We report the longitudinal follow-up of a male child with genetically confirmed BFLS, monitored from the age of 7 months (December 2021) to the age of 4.5 years (October 2025) at the time of manuscript submission, while receiving multidisciplinary rehabilitation. Interventions included physiotherapy targeting postural control and antigravity activation, occupational therapy addressing sensory regulation and purposeful reaching, speech and feeding therapy providing oral-motor stimulation and saliva management, structured caregiver training, and the prescription of assistive devices such as adaptive seating systems and a standing frame. Despite severe axial hypotonia and global developmental delay, the child achieved gradual but modest gains in head control, brief supported sitting, visually guided reaching, and social engagement. Feeding transitioned from mixed oral intake to percutaneous endoscopic gastrostomy (PEG) dependence for nutritional safety and adequacy, while minimal oral stimulation was maintained. A review of the literature confirms that rehabilitation trajectories in BFLS are rarely described and that no standardized guidelines exist. This case contributes one of the few multi-year functional follow-ups available and highlights that early, sustained, and individualized multidisciplinary rehabilitation can promote incremental improvements in motor function, interaction, even in severe presentations, with meaningful implications for quality of life.

## Introduction

Börjeson-Forssman-Lehmann syndrome (BFLS) is a rare X-linked neurodevelopmental disorder first described in 1962 in males with intellectual disability, hypogonadism, obesity, and distinctive craniofacial features [1]. Pathogenic variants in the plant homeodomain finger protein 6 (PHF6) gene, identified in 2002, are responsible for BFLS [2]. The typical phenotype includes moderate-to-severe intellectual disability, axial hypotonia, hypogonadism, and coarse facial features [1,3-5]. Developmental delay is universal, often accompanied by severe language impairment and behavioral variability, reflecting the broad clinical spectrum seen in X-linked intellectual disability syndromes [5,6].

Fewer than 200 cases have been reported worldwide, and the condition is believed to be underdiagnosed due to phenotypic variability and the frequent need for molecular confirmation [3,4,7-9]. Beyond the core dysmorphic and neurological features, BFLS is associated with significant functional impairment from infancy, including marked hypotonia, delayed acquisition of head control, severe limitations in gross and fine motor development, feeding inefficiency, and high dependency in all activities of daily living. Additional manifestations include epilepsy, feeding difficulties, gastroesophageal reflux, ophthalmological anomalies such as strabismus, and variable endocrine or growth abnormalities [3,4,7-9]. Neuroimaging findings such as a thin corpus callosum or a simplified gyral pattern have been described, though they remain inconsistent and nonspecific [3,4]. Familial cases with multiple affected males have been reported, supporting X-linked inheritance and intrafamilial variability [10]. Clinical variability remains substantial, and genotype-phenotype correlations are still incomplete; however, recent reports suggest that some missense variants, including those affecting the plant homeodomain (PHD)-like domain of PHF6, may be associated with milder phenotypes compared with truncating mutations [7-12].

Despite increasing recognition of the genetic and clinical aspects of BFLS, very little is known about longitudinal functional evolution. Published cases rarely provide multi-year developmental data, and rehabilitation outcomes are almost never described in detail [3-6]. Therefore, the functional progression and response to therapy in BFLS remain insufficiently characterized.

We present a genetically confirmed case of BFLS with more than four years of continuous, structured rehabilitation follow-up, providing one of the most detailed longitudinal functional trajectories available and addressing an important gap in current knowledge.

## Case presentation

Infancy (seven months, first evaluation, December 2021): severe axial hypotonia and multisystem involvement

The patient, a male infant born at 39 weeks and 4 days, had a birthweight of 2465 g (small for gestational age), length 49 cm, and head circumference 34 cm. Neonatal complications included respiratory distress, axial hypotonia, feeding dysautonomia, and seizures beginning at three weeks of age, requiring admission to the neonatal intensive care unit (NICU) for 1.5 months (May-June 2021). Early investigations included brain magnetic resonance imaging (MRI) performed in 2021, which demonstrated a thin corpus callosum and a simplified gyral pattern, as well as electroencephalograms (EEGs) showing frequent epileptiform discharges, as documented in official clinical reports. Seizures were managed with antiepileptic therapy under neurology follow-up. Periods of fluctuating somnolence were noted and were likely influenced by both the underlying neurological condition and antiepileptic treatment. Genetic testing confirmed BFLS due to a de novo PHF6 variant (c.729G>A; p.Met243Ile), classified as likely pathogenic according to American College of Medical Genetics and Genomics (ACMG) criteria.

At his first Physical Medicine and Rehabilitation (PMR) evaluation in December 2021 (age 7 months), he presented with severe axial hypotonia, absent head control, poor spontaneous mobility, limited visual engagement, marked fatigability, oromotor inefficiency, and fluctuating somnolence. Physical examination revealed a dysmorphic facial phenotype, including coarse facial features, large and low-set ears, palpebral abnormalities with suspected bilateral ptosis, and divergent gaze. Episodes of abdominal discomfort and hypersensitivity to handling reduced tolerance for prone positioning and play.

He initiated a pediatric rehabilitation program coordinated by a physiatrist, comprising physiotherapy (2-3 sessions/week: cervical activation, postural facilitation, prone tolerance, midline control), occupational therapy (1 session/week: sensory exposure, grasping, early object exploration), and speech therapy (1-2 sessions/week: oral-motor stimulation, saliva management, early feeding skills). Parents received training on positioning, sensory stimulation, and safe feeding strategies. Each session lasted 30-45 minutes, depending on tolerance. During periods of increased seizure activity, irritability, or excessive somnolence, sessions were modified toward shorter, sensory-regulation-focused work prioritizing comfort, alignment, and caregiver guidance rather than motor progression targets.

He was also followed by neurology, gastroenterology, pulmonology, ear-nose-throat (ENT), and endocrinology.

Early toddlerhood (16-22 months, 2022): emerging cervical control and early transitions

By October 2022, the patient demonstrated brief head lifting in prone, improved midline orientation, and occasional rolling into lateral decubitus. Social responsiveness increased, with more reliable visual attention and early directed smiling.

Feeding difficulties persisted, including fatigue during meals and risk of aspiration. Percutaneous endoscopic gastrostomy (PEG) was placed in April 2022 to ensure feeding safety and adequate nutritional intake. A low-profile button was inserted in September 2022. Minimal oral tasting was preserved (2-3 teaspoons) for sensory stimulation.

Rehabilitation emphasized cervical stability, early transitions, supported prone, and seated tolerance. Occupational therapy addressed sensory modulation, toy exploration, and cause-and-effect interaction. Speech therapy focused on saliva control and safe swallowing. Persistent hypotonia led to assessment for adapted seating.

Late toddlerhood (2.5-3.5 years, 2023): improved engagement and prone endurance

At the evaluation in March 2023 (age 22 months), the patient showed intermittent reaching, improved visual engagement, and better differentiated emotional responses. Prone endurance improved, with short periods of cervical and upper-trunk extension. Movements remained poorly coordinated and slow.

PEG remained the primary feeding route, with fluctuating sialorrhea occasionally limiting oral-motor work. Sialorrhea was managed conservatively through speech and feeding therapy strategies and postural adjustments, without pharmacological or invasive interventions. A previous sleep study documented elevated transcutaneous CO₂ levels (median 52 mmHg), supporting the indication for nocturnal non-invasive ventilation; however, this could not be successfully initiated due to poor tolerance, characterized by marked discomfort, hypersalivation, crying, and sleep fragmentation. As a result, it was discontinued and did not become part of the long-term management plan. Poor tolerance to this intervention also limited its integration into the rehabilitation program, particularly given the child’s significant sensory hypersensitivity.

Rehabilitation progressed to proximal stability, supported sitting, and postural reactions. Adaptive seating devices (transport chair, activity chair, bath chair) were prescribed to facilitate alignment and participation at home and school, and a standing frame was introduced for bone health and hip alignment.

Preschool age (4.5 years, 2024-2025): partial head control and brief supported sitting

By June 2024 (age 3 years 11 months), the patient demonstrated partial head control during pull-to-sit, improved prone extension, and attempted to reach toys with the right hand. Sensory hypersensitivities persisted, particularly to sound and touch, occasionally reducing participation. Social interaction increased, with frequent responsive smiling and vocalizations used intentionally to attract attention.

At the January evaluation (age 4 years and 7 months), a mild functional asymmetry was observed, characterized by a preference for the right upper limb and reduced spontaneous use of the left upper limb during play. Neurological examination did not reveal signs of hemiparesis, with symmetrical muscle bulk, preserved passive range of motion, and no focal changes in deep tendon reflexes. By the final October 2025 evaluation (age 4.5 years), he could maintain sitting for approximately one second with anterior hand support and demonstrated consistent social engagement, although motor progress remained limited.

Rehabilitation goals emphasized sitting tolerance, improved postural reactions, and greater environmental interaction. Physiotherapy targeted balance reactions and antigravity control, occupational therapy promoted visually guided reaching and engagement with toys, and speech therapy supported early communication routines. Seating systems were adjusted regularly to optimize posture and comfort. A summary of the patient’s functional evolution is presented in Table 1, and a summary of rehabilitation interventions is presented in Table 2.

**Table 1:** Longitudinal functional evolution of the patient with BFLS (2021-2025). This table summarizes the patient’s longitudinal functional evolution across developmental stages, based on qualitative clinical observation. BFLS, Börjeson-Forssman-Lehmann syndrome; PEG, percutaneous endoscopic gastrostomy

Age/Stage	Gross motor function	Fine motor function	Communication and social interaction	Feeding and oromotor function	Other relevant factors
7 months (Infancy)	Severe axial hypotonia; no head control; poor prone tolerance; minimal antigravity movement	Limited spontaneous hand activity; brief midline contact	Reduced visual engagement; rare vocalizations; occasional social smile	Fatigable oral feeding; poor suck-swallow coordination; drooling	Right cervical preference; plagiocephaly; irritability; tactile hypersensitivity
16-22 months (early toddlerhood)	Brief head lifting in prone; able to roll to lateral decubitus; slight axial tone improvement	Momentary toy contact; emerging bilateral exploration	More consistent social smiling; occasional laughter	PEG placed; small volumes of oral intake are maintained for stimulation	Abdominal discomfort episodes; noise hypersensitivity
2.5-3.5 years (late toddlerhood)	Improved prone tolerance; short periods of cervical extension with support; no independent sitting	Intermittent reaching; momentary grasp; stereotyped movements	Increased engagement, more frequent vocalizations, and better attention to play	PEG-dependent; oral stimulation reduced but preserved; fluctuating drooling	Failed nocturnal ventilation attempts; variable fatigability
4.5 years (preschool age)	Partial head control in pull-to-sit; brief supported sitting; no autonomous mobility	Visually guided reaching; right-hand preference; occasional toy-to-mouth	Intentional vocalizations to seek attention; consistent social smiling	PEG dependence; 2-3 spoonfuls orally for sensory pleasure	Mild functional asymmetry with right upper limb preference; persistent sensory hypersensitivity

**Table 2:** Evolution of rehabilitation therapies over time. This table summarizes the main rehabilitation disciplines, therapeutic focus, and caregiver-oriented interventions across developmental stages.

Age/Stage	Physiotherapy	Occupational therapy	Speech and feeding therapy	Caregiver training/supports
7 months (Infancy)	2-3 sessions/week: cervical activation, postural facilitation, prone tolerance, midline control	1 session/week: sensory exposure, early grasping, object exploration	1-2 sessions/week: oral-motor stimulation, saliva management, early feeding skills	Positioning strategies; sensory modulation; safe feeding guidance
16-22 months (early toddlerhood)	Focus on cervical stability, early transitions, and supported prone	Sensory modulation; toy exploration; cause-and-effect interaction	Swallowing safety; saliva control; maintenance of oral sensory stimulation	Feeding strategies after PEG placement; daily positioning routines
2.5-3.5 years (late toddlerhood)	Proximal stability; supported sitting; postural reactions	Purposeful reaching; sensory integration; play interaction	Communicative intent; oral-motor regulation	Use of adaptive seating; standing frame integration at home and school
4.5 years (preschool age)	Antigravity control; balance reactions; sitting tolerance	Visually guided reaching; engagement with toys and environment	Early communication routines; vocalization support	Ongoing adjustment of seating systems; participation facilitation

## Discussion

To our knowledge, this case provides one of the few longitudinal descriptions of functional evolution in BFLS under structured rehabilitation, covering more than four years of follow-up. The majority of published BFLS reports focus primarily on genetic mechanisms and phenotypic variability [1-9,11,12], whereas long-term functional outcomes, developmental trajectories, and rehabilitation strategies are only minimally documented in the available literature [3-5,7-9].

When compared with previously reported BFLS cases, the developmental trajectory of this patient is consistent with the severe end of the phenotypic spectrum described in the literature. Profound axial hypotonia, markedly delayed motor acquisition, early feeding difficulties, and global developmental delay are frequently reported features [3-5,7-9], and were all present in this child. Epilepsy, common in BFLS with onset in infancy, was also observed early, yet, similarly to published cohorts, seizures improved over time with optimized medical management. Neuroimaging abnormalities such as a thin corpus callosum and simplified gyral pattern, although not diagnostic, have also been documented in earlier series [3,4]. Thus, the overall clinical and neuroimaging constellation in this case aligns well with prior descriptions, although the mild functional asymmetry observed was not associated with any focal or lateralized structural abnormalities on MRI.

Comorbidities played a major role in shaping functional evolution. Severe axial hypotonia limited early motor exploration and delayed acquisition of head control. Feeding difficulties, later requiring PEG placement, reduced opportunities for oral-motor skill development and contributed to fluctuating endurance. Periods of irritability, abdominal discomfort, sialorrhea, and sensory hypersensitivity-although rarely emphasized in BFLS publications-likely interfered with participation and tolerance to therapy sessions. Nevertheless, consistent rehabilitation allowed the child to make incremental gains, including partial head control, brief supported sitting, purposeful reaching, and increased social engagement. These improvements parallel the modest developmental progress described in isolated reports of early intervention in BFLS, suggesting that structured rehabilitation may help optimize functional potential even in severe phenotypes.

This case illustrates important clinical implications for rehabilitation practice in rare X-linked neurodevelopmental syndromes. First, progress may be slow but meaningful, requiring long-term, individualized programs delivered by multidisciplinary teams. Second, comorbidity management, including oromotor, sensory, respiratory, and gastrointestinal issues, is essential to maximize engagement and functional gains. Third, frequent reassessment and equipment adaptation (e.g., seating systems, standing frames) are crucial in children with persistent hypotonia and postural instability.

There is a clear need for systematic approaches to functional assessment in BFLS. Most published cases rely on descriptive developmental summaries rather than standardized measures. The absence of validated outcome tools or multicenter datasets severely limits the ability to establish prognostic patterns. Future research should include longitudinal multicenter registries, standardized functional metrics (e.g., Pediatric Evaluation of Disability Inventory (PEDI) and Gross Motor Function Measure (GMFM)), and structured reporting of rehabilitation interventions to better define expected trajectories and guide clinical decision-making.

In summary, this case adds rare longitudinal evidence that structured, individualized rehabilitation can support gradual yet meaningful developmental progress in BFLS, particularly in domains of interaction, communication, and proximal motor control. Longitudinal documentation remains essential to improve clinical guidance and support the development of future evidence-based rehabilitation recommendations for this rare disorder, highlighting the wider need for multicenter registries and standardized outcome tracking in BFLS.

## Conclusions

To our knowledge, this case provides one of the few multi-year functional descriptions of BFLS, demonstrating that structured, multidisciplinary rehabilitation can support gradual and domain-specific developmental gains despite severe neurodevelopmental impairment. Improvements were most evident in social engagement, communicative intent, and postural tolerance, whereas autonomous gross motor function remained markedly limited. These observations are based on within-patient longitudinal change and should be interpreted within the constraints of a single-case, observational report. Nevertheless, documenting long-term functional trajectories in rare neurodevelopmental syndromes such as BFLS is clinically valuable for counseling families, setting realistic rehabilitation goals, and generating hypotheses for future multicenter studies using standardized outcome measures.

## References

[1] BO M, FO H, LE O (1962). An X-linked, recessively inherited syndrome characterized by grave mental deficiency, epilepsy, and endocrine disorder. Acta Med Scand.

[2] Lower KM, Turner G, Kerr BA (2002). Mutations in PHF6 are associated with Börjeson-Forssman-Lehmann syndrome. Nat Genet.

[3] Turner G, Lower KM, White SM (2004). The clinical picture of the Börjeson-Forssman-Lehmann syndrome in males and heterozygous females with PHF6 mutations. Clin Genet.

[4] Zweier C, Kraus C, Brueton L (2013). A new face of Borjeson-Forssman-Lehmann syndrome? De novo mutations in PHF6 in seven females with a distinct phenotype. J Med Genet.

[5] (2017). Prevalence and architecture of de novo mutations in developmental disorders. Nature.

[6] Gécz J, Shoubridge C, Corbett M (2009). The genetic landscape of intellectual disability arising from chromosome X. Trends Genet.

[7] Crawford J, Lower KM, Hennekam RC (2006). Mutation screening in Borjeson-Forssman-Lehmann syndrome: identification of a novel de novo PHF6 mutation in a female patient. J Med Genet.

[8] Jain V, Foo SH, Chooi S (2023). Börjeson-Forssman-Lehmann syndrome: delineating the clinical and allelic spectrum in 14 new families. Eur J Hum Genet.

[9] Gerber CB, Fliedner A, Bartsch O (2022). Further characterization of Borjeson-Forssman-Lehmann syndrome in females due to de novo variants in PHF6. Clin Genet.

[10] Ernst A, Le VQ, Højland AT (2015). The PHF6 mutation c.1A>G (p.Met1Val) causes Börjeson-Forssman-Lehmann syndrome in a family with four affected boys. Mol Syndromol.

[11] Liu Z, Li F, Ruan K, Zhang J, Mei Y, Wu J, Shi Y (2014). Structural and functional insights into the human Börjeson-Forssman-Lehmann syndrome-associated protein PHF6. J Biol Chem.

[12] Zhang C, Mejia LA, Huang J (2013). The X-linked intellectual disability protein PHF6 associates with the PAF1 complex and regulates neuronal migration in the mammalian brain. Neuron.

